# Trends in medicines procurement by the Brazilian federal government from 2006 to 2013

**DOI:** 10.1371/journal.pone.0174616

**Published:** 2017-04-07

**Authors:** Tatiana Chama Borges Luz, Claudia Garcia Serpa Osorio-de-Castro, Rachel Magarinos-Torres, Bjorn Wettermark

**Affiliations:** 1 René Rachou Research Center/ Oswaldo Cruz Foundation, Belo Horizonte, Minas Gerais, Brazil; 2 Department of Medicine, Clinical Epidemiology, Karolinska Institutet, Stockholm, Sweden; 3 Department of Pharmaceutical Policies and Pharmaceutical Services (NAF), Sergio Arouca National School of Public Health/Oswaldo Cruz Foundation, Rio de Janeiro, Rio de Janeiro, Brazil; 4 School of Pharmacy, Federal Fluminense University, Niterói, Rio de Janeiro, Brazil; 5 Department of Healthcare Development, Public Healthcare Services Committee, Stockholm County Council, Stockholm, Sweden; UNAIDS, GUYANA

## Abstract

The costs of medicines pose a growing burden on healthcare systems worldwide. A comprehensive understanding of current procurement processes provides strong support for the development of effective policies. This study examined Brazilian Federal Government pharmaceutical procurement data provided by the Integrated System for the Administration of General Services (SIASG) database, from 2006 to 2013. Medicine purchases were aggregated by volume and expenditure for each year. Data on expenditure were adjusted for inflation using the Extended National Consumer Price Index (IPCA) for December 31, 2013. Lorenz distribution curves were used to study the cumulative proportion of purchased therapeutic classes. Expenditure variance analysis was performed to determine the impact of each factor, price and/or volume, on total expenditure variation. Annual expenditure on medicines increased 2.72 times, while the purchased volume of drugs increased 1.99 times. A limited number of therapeutic classes dominated expenditure each year. Drugs for infectious diseases drove the increase in expenditures from 2006 to 2009 but were replaced by antineoplastic and immunomodulating agents beginning in 2010. Immunosuppressants (L04), accounted for one third of purchases since 2010, showing the most substantial growth in expenditures during the period (250-fold increase). The overwhelming price-related increase in expenditures caused by these medicines is bound to have a relevant impact on the sustainability of the pharmaceutical supply system. We observed increasing trends in expenditures, especially in specific therapeutic classes. We propose the development and implementation of better medicine procurement systems, and strategies to allow for monitoring of product price, effectiveness, and safety. This must be done with ongoing assessment of pharmaceutical innovations, therapeutic value and budget impact.

## Introduction

Medicines represent one of the largest and fastest growing costs for healthcare systems worldwide. According to data from 161 WHO Member States from 1995 to 2006, per capita spending on pharmaceuticals has increased by approximately 50%, and these increments were more pronounced in middle-income countries, where pharmaceutical expenditure in 2006 was1.76 times greater than in 1995 [1].

Ensuring and expanding access to quality medicines at affordable prices, with adequate financing require an effective healthcare supply system. Ideally, this system should include the selection of reliable suppliers of assured-quality products and the procurement of safer and more cost-effective medicines in the right quantities at the lowest possible total cost to the system optimal and timely delivery [2].

Ageing populations and increasing expenditures on new drugs place considerable pressure on healthcare systems in their efforts to continue to provide comprehensive care [3]. Consequently, not only should cost-effectiveness analyses for the introduction of all new medicines must be made, but new models for the introduction of expensive medicines are needed, as well as effective management of essential medicines for treating common diseases like hypertension, diabetes, and respiratory, cardiovascular and infectious diseases in general [4]. This requires efficient and transparent procurement procedures.

However, achieving these purposes requires a proper balance between the conflicting policy goals of access to medicines and budget control. A comprehensive understanding of how procurement is being conducted, considering price, volume and treatment regimen, the three main components typically identified as affecting pharmaceutical spending [1, 5–7], provides strong support for the development of effective policies.

Medicines policy has been a key part of Brazil's healthcare system since 1998. In that year, the National Medicines Policy was published, and from then on various additional policy documents and norms were added to the legal framework. Medicines policy in Brazil comprises today a wide berth of regulations that influence industrial policies, all regulatory actions, medicines procurement and availability, and medicines utilization in the health system and by individuals. As an upper middle-income country with one of the largest healthcare systems in the world, committed to universal access to medicines, Brazil faces several challenges. Besides its complex administrative structure regarding medicines procurement procedures—conducted independently by more than 5,500 municipalities, 26 states and the Federal District, the Federal Government, as well as hospitals under indirect public administration—, a large portion of medicines expenditures is out-of-pocket and most private health insurance plans fail to cover medicines [8]. The analysis of medicines expenditures must be performed, thus, for each public level to identify potential problems and formulate adequate and cost-effective policies [9].

Such analyses require access to high-quality data on pharmaceutical expenditure, which has generally been lacking for low- and middle-income countries [1]. This study addresses this gap by examining public medicine procurement in Brazil. Trends in purchases from 2006 to 2013 both in general and by different therapeutic groups were evaluated to identify the key drivers of increases or decreases in spending.

## Methods

### Data source

Data for this study were obtained from the Integrated System for Administration of General Services/*Sistema Integrado de Administração de Serviços Gerais* (SIASG) database. SIASG is a publicly available general procurement database of the Brazilian federal government (http://dados.gov.br/dataset/compras-publicas-do-governo-federal). Our data included medicines purchases by all federal government bodies (Ministries of Health, Defense, Education, and Justice, among others).

We analyzed pharmaceutical purchases from January 2006 to December 2013. These were considered the most consistent data for two reasons: (1) the SIASG registry protocol was updated in 2005 and (2) 2013 was the last full year of available data when the study began. Data were extracted for all medicines. Every purchase is individually described in the database with information on the drug (name, dosage form and strength), unit purchase price, and purchased quantity in number of drug packaging units.

### Analysis

In order to evaluate procurement trends from 2006 to 2013 both in general and by different therapeutic groups and to identify the key drivers of increases or decreases in expenditure, we conducted several analyses.

All pharmaceutical products were classified in accordance with the WHO-ATC/DDD system coordinated by the WHO Collaborating Centre for Drug Statistics Methodology. The ATC system classifies medicines in groups at five levels, starting by fourteen main groups, followed by pharmacological/therapeutic subgroups (2^nd^ level), chemical/pharmacological/therapeutic subgroups (3^rd^ and 4^th^ levels) and the chemical substance (5^th^ level). The ATC classification is used worldwide for comparison of drug consumption and as a tool for drug utilization research[10]. Analyses were conducted at two different ATC levels: anatomical main group (ATC 1^st^ level) and therapeutic groups (ATC 2^nd^ level). Descriptive statistics expressing data as frequencies and percentages were estimated using SPSS 22.0 for Windows (IBM Corporation, USA) and Microsoft Excel 2007.

### Expenditure and volume estimations

Medicine purchases were aggregated by volume (number of drug packaging units purchased) and expenditure (number of drug packaging units purchased multiplied by unit purchase price) for each year from 2006 to 2013.

Data on expenditure were adjusted for inflation using the Extended National Consumer Price Index (IPCA) with December 31, 2013, as the reference date. This index is provided by the Brazilian Institute of Geography and Statistics (IBGE) [11] and is used by the Brazilian Central Bank. Expenditure was measured in US Dollars (USD) (1 USD = 2.3426 Brazilian Reais (BRL), December 31, 2013).

### Lorenz curves

Lorenz curves were used to study the cumulative proportion of drug purchases by therapeutic groups [12]. Purchases were ranked in descending order by expenditure. The cumulative percentage of expenditure was plotted along the vertical axis versus the cumulative percentage of therapeutic classes (ATC 2^nd^ level) purchased on the horizontal axis. Differences in expenditures on purchased therapeutic classes can be identified by visual inspection of the curve. A diagonal line is expected if all therapeutic classes are being purchased in similar amounts, and the curve deviates from this diagonal when it indicates the existence of quantitative differences in spending on each class. We analyzed the Lorenz 1^st^ and 50^th^ percentiles, i.e., the proportions of total expenditure that 1% and 50% of all therapeutic groups accounted for, respectively.

### Expenditurevariance analysis

Expenditure variance analysis is a descriptive method designed to compare performance to budgets (i.e. expected values) in a given time period. The components of an expenditure variance can be divided into two broad elements: price variance and volume variance[13,14].

Considering the medicines purchase process, expenditure variance may be a result of paying more or less than the standard for a given product—price variance component—or of an increase or decrease in the quantities purchased—volume variance component—or of a combination of the two factors [13,14].

To determine the impact of each factor, purchase price and volume, on total expenditure variation from 2006 to 2013, actual and standard average unit purchase prices (AP and SP), were calculated by dividing total expenditure by total volume purchased, aggregated by therapeutic class, for each of the top 20 therapeutic classes. Prices were corrected by the Extended National Consumer Price Index (IPCA) as mentioned previously. Purchased quantity and average unit purchase price from 2006 were considered as standards, SQ and SP, respectively, while data from 2013 provided information on actual quantity purchased (AQ) and actual average unit purchase price (AP).

The price, volume and expenditure variances were computed using the following formulas:

Price variance =(AQ×AP)−(AQ×SP)

Where:

AQ is ‘actual quantity purchased’

AP is ‘actual average unit purchase price’

SP is ‘average unit purchase price’

Volume variance=(AQ×SP)−(SQ×SP)

Where:

AQ is ‘actual quantity purchased’

SP is ‘average unit purchase price’

SQ is ‘purchased quantity’

Expenditure variance=(AQ×AP)−(SQ×SP)

Where:

AQ is ‘actual quantity purchased’

AP is ‘actual average unit purchase price’

SP is ‘average unit purchase price’

SQ is ‘purchased quantity’

### Ethical aspects

The study analyzed publically available data and did not involve human subjects, specimens or tissue samples, or vertebrate animals, embryos, or tissues. There was no need of prior approval by an Institutional Review Board.

## Results

From 2006 to 2013, the Brazilian Federal Government purchased, nearly 23 billion drug packaging units (Table 1) belonging to 15 different anatomical main groups (ATC 1^st^ level) and 90 different therapeutic groups (ATC 2^nd^ level). The total medicine expenditure, adjusted for inflation, was USD 14.7 billion (BRL 34.6 billion) during the period (Table 1). Annual expenditure increased 2.72 times (172%) from 2006 to 2013, while volume increased 1.99 times (100%). The top twenty therapeutic groups accounted for more than 90% of total expenditure and volume each year (Table 1).

**Table 1:** Annual total and by top 20 medicine expenditures and total purchase volume in drug packaging units. Brazil, 2006–2013.

Year	Total expenditureUS$	Top 20 expenditureUS$		Total volume	Top 20 volume	
		US$	(%)		US$	(%)
2006	1,124,158,372	1,052,500,608	93.6	2,070,642,460	1,933,132,953	93.4
2007	1,116,143,137	1,054,406,762	94.5	1,881,582,657	1,700,828,887	90.4
2008	1,126,058,529	1,054,840,245	93.7	3,028,162,544	2,866,179,975	94.7
2009	1,633,804,693	1,521,550,673	93.1	2,008,236,794	1.832.744.729	91.3
2010	1,755,370,595	1,658,063,509	95.5	4,182,515,053	3,863,864,788	92.4
2011	2,653,708,350	2,548,087,364	96.0	2,650,220,185	2,460,961,517	92,9
2012	2,327,737,837	2,223,653,812	95.5	3,054,801,562	2,884,111,304	94,4
2013	3,053,794,154	2,887,769,786	94.2	4,123,525,494	3,700,593,751	89.7
Total	14,790,775,666	14,000,872,759	94.7	22,999,686,749	21,242,417,904	92.4

**Fig 1:**
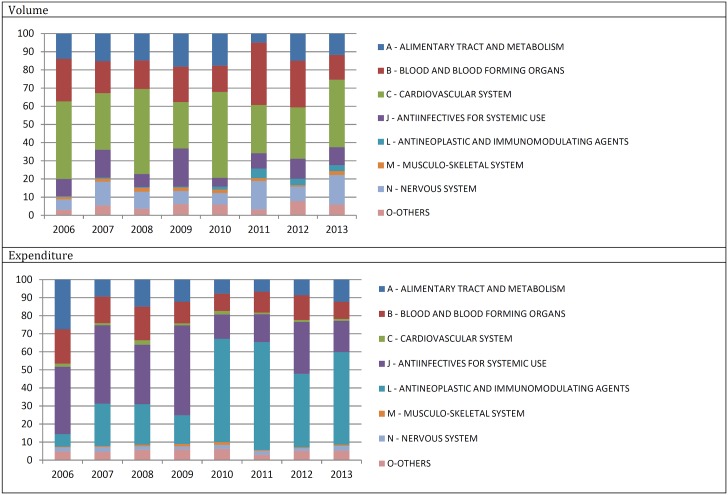
Volume and expenditure by major classes of medicines. Brazil, 2006–2013.

During the period, there was considerable variation in the use of different drug classes. In 2006, cardiovascular drugs (ATC main group C) accounted for the largest proportion of volumes while drugs for treating infectious diseases (ATC main group J) dominated expenditures (Fig 1). In 2013, cardiovascular drugs were the most important class in terms of volume, while antineoplastic and immunomodulating agents (ATC main group L) emerged as the main group in expenditures. Antineoplastic and immunomodulating agents (L) were the therapeutic class with the broadest variation during the period, with a 20-fold increase in expenditure from 2006 to 2013, from USD78.2 million (BRL183.2 million) to USD1.57 billion (BRL3.7 billion).

**Fig 2:**
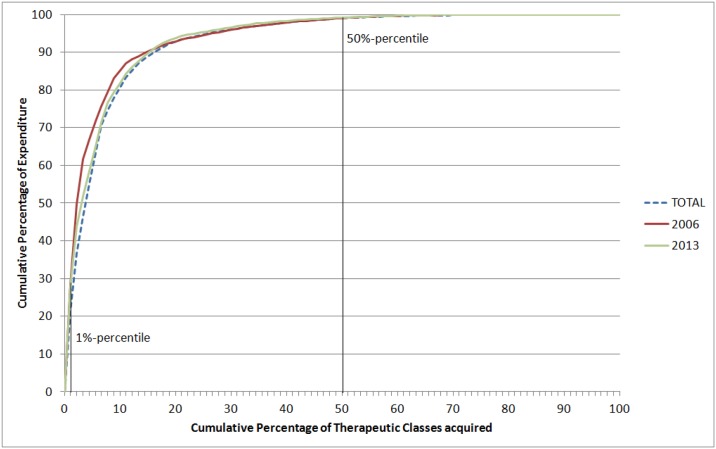
Lorenz curves from Brazilian federal government purchases. Brazil, 2006–2013. Number of therapeutic classes: 90 (ATC 2nd level) 1% Percentiles of Lorenz curves: J05 (2006); L04 (2013; 2006–2013). 50% Percentiles of Lorenz curves: J05, A16 (2006); L04, L01, J05 (2013); L04, J05, L01 (2006–2013).

A limited number of therapeutic areas dominated expenditure each year (Fig 2) and the therapeutic groups constituting the 1% percentile and the 50% percentile of the Lorenz curve only showed minor variation. From 2006 to 2009, antivirals (J05) constituted the 1% percentile whereas from 2010 to 2013, immunosuppressants (L04) became the main class. Any class at the curve’s 1% percentile accounted for approximately thirty percent of total expenditure, regardless of the year. A few therapeutic groups constituted half of total expenditure (50% percentile of the Lorenz curve): antivirals (J05) and various enzymes/amino acids (A16) in 2006 and immunosuppressants (L04), antineoplastic (L01) and antivirals (J05) in 2013. Considering the entire period (2006–2013), just three therapeutic groups (immunosuppressants, antivirals, and antineoplastic agents) dominated expenditure, constituting the curve’s 50% percentile (see also Table 2).

**Table 2:** Top 20 therapeutic classes by annual expenditure, by total expenditure in the period, and by expenditure variation (in USD). Brazil, 2006–2013.

Therapeutic class	2006	2007	2008	2009	2010	2011	2012	2013	Total 2006–2013	Expenditure variation (%)
L04—immunosuppressants	3.4	9.8	14.6	8.6	703.1	956.8	611.9	858.9	3166.9	25098.7^a^
J05—antivirals for systemic use	320.1	208.4	229.7	594.1	84.2	229.5	331.6	243.1	2240.7	-24.0
L01—antineoplastic agents	43.7	81.2	82.5	105.9	152.6	349.0	164.3	478.4	1457.6	993.6^b^
B02—anti-hemorrhagic agents	134.8	101.9	126.4	135.2	116.5	243.4	234.7	192.6	1285.5	42.9
L03—immunostimulants	22.3	158.4	143.7	129.8	134.1	264.0	157.2	204.9	1214.4	819.4^c^
A16—other alimentary tract and metabolism products	238.7	7.4	109.4	149.3	92.6	161.4	123.4	198.5	1080.7	-16.8
J07—vaccines	0.2	175.6	6.0	94.5	51.0	8.3	199.6	45.9	581.2	19347.4^d^
A10—drugs used in diabetes	54.9	86.4	48.7	32.0	30.2	5.3	62.6	158.8	478.8	189.3^e^
J06—immune sera and immunoglobulins	43.0	44.2	71.5	46.9	12.2	88.5	12.8	88.9	408.1	106.8^f^
J01—antibacterials for systemic use	42.2	32.9	34.6	53.0	54.8	50.2	59.5	75.0	402.4	77.7
J02—antimycotics for systemic use	9.5	20.5	26.6	23.8	32.5	32.5	62.9	70.4	278.7	639.8^g^
B05—blood substitutes and perfusion solutions	13.3	24.4	33.1	39.2	35.8	40.4	28.7	57.1	272.0	329.7^h^
G03—sex hormones and modulators of the genital system	3.6	22.1	25.0	5.8	38.7	19.8	26.2	39.7	180.8	1013.2^i^
B03—antianemic preparations	57.6	28.7	36.0	1.5	1.4	2.3	32.6	3.5	163.6	-94.0
V03—all other therapeutic products	1.1	1.5	2.5	22.7	29.5	3.4	47.2	45.2	153.0	3997.8^j^
B01—antithrombotic agents	8.4	9.3	14.4	17.7	15.6	20.6	21.3	36.0	143.3	331.0^k^
L02—endocrine therapy	8.8	12.5	11.6	14.5	13.8	14.4	13.0	27.2	115.9	210.1^l^
N01—anesthetics	7.4	13.4	8.3	13.2	10.9	15.7	14.0	20.2	103.2	174.8^m^
V08—contrast media	4.0	6.7	14.3	13.4	8.5	10.6	7.7	8.6	73.7	117.3^n^
C10—lipid modifying agents	5.2	2.2	9.1	2.0	13.6	9.9	10.8	8.3	61.3	59.0

Expenditures are presented in USD million (1 USD = 2.343 BRL)

Expenditure variation = **(2013–2006)/2006**

Percentages higher than 100% can also be described in terms of "x fold increase". Converted percentages were rounded off accordingly:

^a^: 250 fold increase;

^b^: 9.9 fold increase;

^c^: 8.2fold increase;

^d^: 193 fold increase;

^e^: 1.9 fold increase;

^f^: 1.1 fold increase;

^g^: 6.4 fold increase;

^h^: 3.3 fold increase;

^i^: 10 fold increase

^j^: 40 fold increase;

^k^: 3.3 fold increase;

^l^: 2 fold increase;

^m^: 1.7 fold increase;

^n^: 1.2 fold increase

Table 2 shows the top 20 therapeutic classes in terms of expenditure, purchased from 2006 to 2013. These classes came from eight main groups (A, B, C, G, J, L, N, and V). While most classes (n = 17) showed an increase in expenditure, ranging from 43% for anti-hemorrhagic agents to 250 times for immunosuppressants (25098.7%), the reduction in spending in the remaining three classes varied from 16.8% (alimentary tract and metabolism) to 94% (other therapeutic groups).

Table 3 shows the results of expenditure variance analysis. For the majority of the therapeutic classes (n = 11), price variance was the main factor explaining expenditure variance during the period 2006–2013.

**Table 3:** Price and volume variances and results of expenditure variance analysis by therapeutic subgroup. Brazil, 2006–2013.

Therapeutic subgroup	Price variance^a^		Volumevariance^b^		Expenditure variance^c^
	Value	%	Value	%	
L04—immunosuppressants	630.8	73.7	224.7	26.3	855.4
J05—antivirals for systemic use	-644.8	838.2	567.8	-738.2	-76.9
L01- antineoplastic agents	238.7	54.9	195.9	45.1	434.6
B02-anti-hemorrhagic agents	52.9	91.5	4.9	8.5	57.9
L03- immunostimulants	-282.8	-154.9	465.4	254.9	182.6
A16—other alimentary tract and metabolism products	-33.1	82.4	-7.1	17.6	-40.2
J07—vaccines	-56.0	-122.6	101.6	222.6	45.7
A10—drugs used in diabetes	41.7	40.2	62.2	59.8	103.9
J06—immune sera and immunoglobulins	-114.2	-248.7	160.1	348.7	45.9
J01- antibacterials for systemic use	26.9	82.1	5.9	17.9	32.8
J02 antimycotics for systemic use	59.9	98.5	0.9	1.5	60.9
B05—blood substitutes and perfusion solutions	-4.1	-9.3	47.9	109.3	43.8
G03—sex hormones and modulators of the genital system	-159.5	-441.6	195.6	541.6	36.1
B03—antianemic preparations	-121.0	223.7	66.9	-123.7	-54.1
V03—all other therapeutic products	-202.9	-460.6	247.0	560.6	44.0
B01—antithrombotic agents	27.9	100.8	-0.2	-0.8	27.7
L02—endocrine therapy	11.1	60.2	7.3	39.8	18.4
N01—anesthetics	0.2	1.7	12.6	98.3	12.9
V08—contrast media	-4.8	-103.9	9.5	203.9	4.6
C10—lipid modifying agents	3.2	104.1	-0.1	-4.1	3.1

Expenditures are presented in USD million (1 USD = 2.343 BRL).

AQ = actual quantity purchased; AP = actual average unit price; SQ = standard quantity purchased; SP = standard average unit price

^a^(*AQ*_2013_ x *AP*_2013_)—(*AQ*_2013_ x *SP*_2006_);

^b^(*AQ*_2013_ x *SP*_2006_)—(*SQ*_2006_ x *SP*_2006_);

^c^(*AQ*_2013_ x *AP*_2013_)—(*SQ*_2006_ x *SP*_2006_)

Price and volume varied in the same direction for nine therapeutic classes. Of those, seven classes (L04, L01, B02, A16, J01, J02, and L02) showed price variance as the primary factor contributing to expenditure. For example, the main reason for the increase in spending on immunosuppressants (L04), antineoplastic agents (L01), and antimycotics for systemic use (J02) during the period was price variation, which explained 73.7%, 55.0%, and 98.5% of expenditure variance, respectively.

For two therapeutic classes (A10, N01), volume variance was the main driver of expenditure variance. In the case of A10 drugs (used in diabetes), volume variance accounted for almost 60% of expenditure variance (nearly USD104 million).

Eleven therapeutic classes showed price and volume variances in opposite directions (Table 3). For three therapeutic classes (J05, B03, B01), the effect of price reduction was nearly counterbalanced by volume variance, but still prevailed. For class C10 (lipid modifying agents), there was a volume reduction, but the price increased over time. For the remaining seven classes (J07, L03, J06, B05, G03, V03, V08), volume variance attenuated the effect of price decrease over time.

## Discussion

Our findings suggest a significant increase in pharmaceutical expenditure by the Brazilian Federal Government from 2006 to 2013. Total expenditure practically tripled, while the quantities purchased only doubled during this eight-year period. This tendency of growth in pharmaceutical spending is being observed worldwide, but at different rates [15–19]. In Canada, from 2006 to 2011, government medicines expenditures increased at an average annual rate of 4.5% [17], while in Brazil the rate was 13.3% per year. In China, a similar (14.9%) yet non-deflated annual growth rate for total drug expenditure was observed for the period 1990–2009 [18].

The main factors that can drive up expenditure on pharmaceuticals in a country are high purchase prices, high-use patterns (i.e. quantity), or a combination of the two [1, 5–7]. Switches to new therapies and an adopted high-priced treatment regimen, as in Brazil, are definite explanatory factors. This is corroborated by reports from the National Committee for Health Technology Incorporation (CONITEC, formerly CITEC). According to these reports, 60% of medicines incorporated by the Brazilian Health System (SUS) from 2012 to 2016 were high-priced products [20].

A few classes dominated expenditures during the period in question, and price-related determinants strongly affected drug expenditure. Despite variation in the use of different drug classes, a common issue was the replacement in the ranking over time, of less expensive medicines, such as those for treating infectious diseases (J), with newer, more costly and patent-protected medicines, such as antineoplastic and immunomodulating agents (L). These results are in line with findings from other countries [21, 22].

Immunosuppressants (L04) include anti-TNF-α (tumor necrosis factor alpha) drugs and interleukin inhibitors. These drugs, used to treat rheumatoid arthritis (worldwide prevalence rate estimated at 0.5–1.0% of the population) and Crohn’s disease (estimated incidence rate 6.3/100,000 person-years) [17, 23, 24], showed the most substantial expenditure increase, with more than 250 times (25098.7%), accounting for one third of purchases since 2010. For this class, price was the main factor boosting expenditure. Many high-priced new medicines were introduced in this class. This finding corroborates previous literature. Anti-TNF drugs were one of the classes that most contributed to the growth of public drug spending in Canada between 2007 and 2012, reaching 54.8% [17].

The overwhelming price-related increase caused by L04 class is bound to have an important impact on the sustainability of Brazil’s pharmaceutical supply system. If price reduction strategies are not implemented, Brazil’s selection, incorporation, and purchase of high-priced innovative drugs, e.g., the recently adopted direct acting antivirals (DAAs) for hepatitis C (sofosbuvir, daclastavir, and simeprevir), may challenge the continuity of provision, given the same level of financing, if other access mechanisms such as generic production cannot be achieved [25]. Furthermore, the new medicines are under patent protection, and competition is limited or nonexistent [20, 26, 27].

Volume was the main driver of expenditure for a few therapeutic classes, such as immunostimulants, vaccines, drugs used in diabetes, sex hormones, modulators of the genital system, and anesthetics. These results may reflect an increase in coverage, whereby more patients can take advantage of the provision of these medicines. Such gains may reflect either economies of scale in procurement procedures, therapeutic reference pricing, or generic substitution. Part of this increase may also be explained by the introduction of new medicines to treat previously untreatable diseases within these classes [22].

In general, confronting the great concentration of purchases on a few therapeutic classes within the Brazilian burden of disease suggests that the federal government should reorient medicines procurement policies and strategies. Technology adoption in the country, presently led by the National Committee for Health Technology Incorporation (CONITEC), might profit by focusing more on the country’s epidemiological profile, where leading causes such as cardiovascular diseases, mental disorders (particularly depression), diabetes, and chronic obstructive pulmonary disease account for more than 50% of the disease burden [28].

The main purpose of this article was to estimate medicine expenditure by the Brazilian Federal Government and examine procurement trends from 2006 to 2013. This was the first study to analyze expenditure by therapeutic classes and to disaggregate the drivers of spending growth or decline within each of the major classes.

While some cost-management strategies were adopted by the Brazilian government during the period in question, the study illustrates some of their potential pitfalls. CONITEC produces therapeutic protocols and a federal medicines list, which are, by law, the basis for medicines financing and prescription in the public healthcare system. Notwithstanding, physicians throughout the system make individual therapeutic choices that impact expenditures. The medicines selection process is not accepted by all and suffers greatly from pressures for innovation [29].

Other factors may also be involved, from lack of the necessary purchaser’s power in price negotiations [1, 30–32], as befalls many other financially constrained middle-income countries, to inadequate revision and faulty adherence to therapeutic guidelines [1].

We presented a detailed picture of medicine purchases by the Brazilian federal government. Although the article addresses trends in medicines procurement over time, some caveats deserve consideration. First, SIASG is a national database that registers medicine purchases and does not allow an assessment of dispensed/prescribed medicines at the individual patient level, so the estimates are a proxy for actual consumption. Considering the complexity of the administrative structure for medicine procurement procedures in Brazil, it is presumed that these analyses represent about one third of drug procurement in the country [9, 33], a considerable amount. Additionally, SIASG does not offer any margins or mark-up values, such as distribution margins, service fees etc. Therefore we cannot estimate costs for the healthcare system, only direct drug expenditures (volume x price). In market competition situations, significant differences between drug price and drug production cost can considerably burden health system expenditures[34].

We classified medicines according to the Anatomical Therapeutic Chemical (ATC) classification. However, we could not assign a Defined Daily Dose(DDD) for some medicines for which there are no available DDDs, thus limiting comparisons. Importantly, we did not analyze individual medicinal products, and our interpretation of the observed changes presumed utilization by aggregated therapeutic classes over time.

Nevertheless, we believe our results are valuable for elucidating drug procurement practices by the Brazilian Federal Government’and consequently by the entire public sector in the country, which is all subject to binding legislation and regulations. SIASG is a large federal procurement database that can be used for planning and forecasting. Given the trends in expenditures and the rise within specific therapeutic classes, it is important that guidance in drug procurement strategy be developed and implemented, including systems that allow monitoring product price, effectiveness, and safety, as well as assessment of pharmaceutical innovations, therapeutic value, and budget impact.
